# Radiological Imaging of Gastro-Entero-Pancreatic Neuroendocrine Tumors. The Review of Current Literature Emphasizing the Diagnostic Value of Chosen Imaging Methods

**DOI:** 10.3389/fonc.2021.670233

**Published:** 2021-06-15

**Authors:** Gabriela Półtorak-Szymczak, Tadeusz Budlewski, Mariusz Ireneusz Furmanek, Waldemar Wierzba, Katarzyna Sklinda, Jerzy Walecki, Bartosz Mruk

**Affiliations:** ^1^ Department of Radiology, Centre of Postgraduate Medical Education, Warsaw, Poland; ^2^ Central Clinical Hospital of the Ministry of the Interior and Administration in Warsaw, Warsaw, Poland; ^3^ Department of Nuclear Medicine, Central Clinical Hospital of the Ministry of the Interior and Administration in Warsaw, Warsaw, Poland; ^4^ University of Humanities and Economics, Lodz, Poland

**Keywords:** gastro-entero-pancreatic neuroendocrine tumor, magnetic resonance imaging, computed tomography, ultrasonography, radiology, neuroendocrine tumor

## Abstract

Despite development of radiologic imaging, detection and follow-up of neuroendocrine neoplasms (NENs) still pose a diagnostic challenge, due to the heterogeneity of NEN, their relatively long-term growth, and small size of primary tumor. A set of information obtained by using different radiological imaging tools simplifies a choice of the most appropriate treatment method. Moreover, radiological imaging plays an important role in the assessment of metastatic lesions, especially in the liver, as well as, tumor response to treatment. This article reviews the current, broadly in use imaging modalities which are applied to the diagnosis of GEP-NETs, (the most common type of NENs) and put emphasis on the strengths and limitations of each modality.

## Introduction

The diagnostic and therapeutical process of gastro-entero-pancreatic neuroendocrine tumors(GEP-NETs) has derived a lot of difficulties for many years, and requires multidisciplinary cooperation to make the most appropriate diagnosis, and choose the best treatment. The vast range of radiologic imaging methods could be used in the tumor assessment and staging, e.g., ultrasonography, computed tomography and magnetic resonance. By using different modalities, it is possible to collect information about the biology of tumor tissue (1–3), and find secondary lesions; the most common site of metastases is the liver, which makes the imaging of this organ crucial (4). A role of nuclear medicine for diagnosing and treatment of GEP-NETs is noteworthy, also (2).

In recent years, an increasing trend in morbidity is observed which could be credited to the development of diagnostic imaging and a higher awareness of the possibility of occurrence of GEP-NETs (5–7).

For better communication, the World Health Organization (WHO) proposed changes in NENs classification in 2017. NENs were divided into three groups: well differentiated NENs, poorly differentiated NENs and mixed neuroedncorine non-endocrine neoplasm (MiNEN). The group of well differentiated NENs stands for NETs which are divided into three subgroups: grade one to three (G1-G3), and poorly differentiated NENs stands for NEC grade three (G3) (8).

## Survey Methodology

The authors searched the literature of interest using Google Scholar and PubMed with the following key words: neuroendocrine tumors, imaging GEP-NET, GEP-NEN; abdominal MRI, abdominal CT, nuclear medicine, ultrasonography; endoscopic ultrasonography and PET. Literature reviews, original articles and guidelines were included in the search.

Firstly, abstracts of 80 articles were read and analyzed, then authors selected 63 the most comprehensive papers and based on them. The majority of articles was submitted in last 5 years.

## Discussion

Ultrasonography(US) and computed tomography(CT) are still used as elementary modalities in primary diagnosis of GEP-NETs; they allow to depict morphological features of lesions and estimate severity of the disease. Additionally, magnetic resonance imaging (MRI) applications could approach to functional attributes (9).

### Ultrasonography

Transabdominal ultrasonography (US) is broadly available and it is usually the first imaging test performed. According to *Diagnostic and therapeutic guidelines for gastro-entero-pancreatic neuroendocrine neoplasms*, US has great value in assessment of the liver metastases (sensitivity 85–90%); by contrast, the sensitivity of this method in the diagnosis of pancreatic tumors is only 13% to 27%. Moreover, above mentioned, *Diagnostic and therapeutic guidelines for gastro-entero-pancreatic neuroendocrine neoplasms* does not find *US* useful in the assessment of other parts of the gastrointestinal tract (10).

The drawbacks of this modality are differences in sensitivity, depending on anatomical conditions, cooperation with the patient, and experience of the physician. These conditions could rule out satisfying results (10).

The US results could be improved by use of contrast medium, and is called contrast enhanced ultrasonography (CEUS). CEUS is enriched with intravenous contrast administration—a suspension of microbubbles stabilized by phospholipids and containing sulphur hexafluoride (11). Its sensitivity is about 99%. In CEUS imaging, the enhancement of a tumor could be assessed in the same way as in computed tomography, in arterial, parenchymal, and late venous phases (12, 13).

Moreover, similarly to CT, CEUS could detect secondary to treatment changes, like necrosis. CEUS could be used in follow-up liver and pancreatic lesions which leads to limiting multiple iodine contrast administration or X-ray exposure (14). The next application of ultrasonography is an endoscopic ultrasonography (EUS), a procedure, which has high sensitivity in assessment of pancreatic lesions. The ENETS Consensus Guidelines proposed EUS (sensitivity from 57% to 94%) as a modality of choice after other negative non-invasive imaging studies. EUS could predict the grade of tumor differentiation and consequently points a physicians toward the best method of treatment (surgical or endoscopic). However, its accuracy depends on the skills of the physician and the distance between the probe and lesion (15).

EUS enables the detection of gastric NENs with diameters less than 10 mm and duodenal NENs (16). The appearance of NEN on EUS is similar to the image obtained in transabdominal US. Importantly, high-grade NEN could be more difficult to distinguish from pancreatic adenocarcinoma (15, 17).

In comparison to CT, EUS has a great value in detecting insulinoma (sensitivity 71-94%). CT scan sensitivity was almost three times lower in this case (20-63%) (18). Moreover, in EUS procedure, it is possible to conduct fine needle aspiration (FNA) and obtain a specimen for histopathological examination (19).

### Computed Tomography

Computed tomography (CT) is higly avaiable and it is an elementary imaging technique, with relatively short acquisition time. In the context of NEN imaging, CT is used to determine the origin, staging, and monitoring treatment effects. It is crucial to choose a proper protocol; firstly, to emphasize the characteristic radiologic features of GEP-NETs-strong enhancement in the arterial phase, secondly to separate the phases for the most accurate evaluation of metastasis (20).

The above mentioned CT protocol should consist of scans in pre- and postcontrast phases. The scans in native phase could reveal calcifications, while the analysis of postcontrast pictures derives information about enhancement and wash out. The optimal contrast agent flow is required to be at least 3 to 4 ml/s (21). It is important to appropriately assess morphologic features and localization in relation to bile or pancreatic ducts and vessels. Moreover, morphological features, such as large diameter (>3 cm)/large volume, foci of necrosis, and infiltration of adjacent organs could correlate with the tumor grade (10, 22). The studies focusing on pancreatic NET(PNET), conducted by Kim et al. and Park et al., proved that using contrast enhanced computed tomography (CECT) could differentiate between G1/G2 and G3 tumors (23, 24). In addition, the CT features, e.g., tumor size, correlate with both, the mitotic count and Ki-67 index which associated with tumor homogeneity (22).

CT is the best modality for assessing vessel infiltration and has a high value before planning for surgery. CT can help with differentiation between benign and malignant pancreatic lesions, such as mass forming panceratitis (MFP) and non-hypervascular NET. The non-hypervascular PNETs have better defined margins and CT enhancement values are lower in the arterial and venous phases than in the MFP (25).

According to *Diagnostic and therapeutic guidelines for gastro-entero-pancreatic neuroendocrine neoplasms*, the sensitivity of CT examination in the diagnosis of pancreatic tumors is 73% (63%–82%), and specificity is 96% (83%–100%). Moreover, CT has high sensitivity and specificity in detecting metastases, for example, in case of hepatic metastases the sensitivity is 82% and the specificity 92%, in case of lymph nodes metastases sensitivity 60% to 70% and specificity 87% to 100% (10, 26).

Compared to MRI, CT is preferred for imaging of the lungs, as it offers a better spatial resolution and it is considered to be the most effective tool for detecting small bowel NETs (18, 27). The drawback of CECT is its low sensitivity in the detection of lesions smaller than 1 cm and bone metastasis-sensitivity 58% (20, 28).

CT enteroclysis has a sensitivity about 92%, and a positive predictive value of almost 95% (29–31). According to the *ENETS consensus guidelines update for neuroendocrine neoplasms of the jejunum and ileum*, enteroclysis is beneficial for assessing the small bowel, in case of failure of CT scan in the localization of the primary tumor. The patient’s preparation is crucial for the CT enteroclysis protocol and consists of application of neutral contrast medium (water and methylcellulose) by a nasojejunal tube before acquisition, and optional administration of spasmolytics (29). According to study by Paulsen at al., CT enteroclysis with optimum distention of the bowel lumen can increase the detection of small mucosal tumors (32). However, bowel palpation during surgery could not be replaced (33).

The protocol consists of two phases 25 s and 60 s after the start of intravenous contrast injection. CT enteroclysis allows radiologists to assess extrajejunal diseases, such as: desmoplastic reaction, fibrosis, metastases in solid organs, and detection of metastatic enlarged lymph nodes (29). CT enterography is similar to CT enteroclysis, the only difference is the method of contrast administration – before CT enterography a patient drinks about 1L of oral negative contrast. The sensitivity of both methods is comparable (32). An additional information may also be obtained with a capsule endoscopy, which may identify lesions in approximately 50% of cases.

Owing to its broad availability and short scanning time, CT is an excellent method for detecting complications secondary to GEP-NETs, such as bowel obstruction, intussusception, or desmoplastic reaction, which are life-threatening diseases (34).

The application of Dual Energy Computed Tomography (DECT) in routine examination and follow-up in patients with NET is rare. However, Noah et al. proved that CT iodine maps could raise radiologist confidence (35). DECT differentiates tissue specimens by using different X-ray spectra, for example 80 and 140 kV, simultaneously without increasing patient radiation dose and, as a result, derives images with higher contrast between the lesion and surrounding tissue (35, 36).

### Magnetic Resonance Imaging

Magnetic resonance imaging (MRI) is a modality that allows to assess a neoplasm, similar to CECT. According to the *ESGAR consensus* statement, CE-MRI is recommended for routine imaging (37).

The standard, abdominal MRI protocol contains the following sequences: T1-weighted pre- and post-contrast, T2-weighted, in-phase and opposed phase, fat-suppressed T2-weighted; gradient echo and spin echo diffusion-weighted images (DWI) sequence with corresponding apparent diffusion coefficient (ADC) map, which includes both morphological and functional features of the lesion. The MR protocol could be extended by cholangiopancreatography sequences, which could be very useful in the imaging of PNETs (38, 39). Dynamic or multiphase contrast enhanced acquisitions should be performed with breath holding.

MRI is considered to be the best in detecting and characterizing metastatic liver lesions (2, 10).

Radiologists conducting MRI could choose liver-specific contrast agents that provide better visualization of focal liver lesions and a high contrast-to-noise ratio. Tirumani et al. proved that the late hepatocellular phase is the best for measuring lesion size (40).

On the other hand, Dormain et al. proved that scans obtained in the arterial phase are most appropriate for the detection of hepatic metastases (41). In addition, Ronot et al. proved that postcontrast sequences have higher sensitivity in detecting metastatic lesions than pre-contrast sequences (42), which corresponds with the article by Tirumani (40).

Additionally, pre- and postcontrast scans allow to differentiate between benign and malignant lesions (43). MRI allows to distinguish non-hyperfunctioning NET (NF-NET) and adenocarcinoma; NF-NETs are hyperintense lesions in T2 and present vivid arterial enhancement, while adenocarcinomas are hypointense on all pulse sequences and do not show arterial enhancement (39).

Radiologists should be aware of some pitfalls during CE-MRI. Firstly, the typical enhancement in the arterial phase and lack of enhancement after intravenous contrast administration in the hepatobiliary phase are not specific for NET only. In cases of doubt, PET imaging with Ga-68-DOTANOC tracer should be considered (44). Secondly, in hepatobiliary phase, there is a possibility of enhancement of liver metastasis, which is caused by the accumulation of contrast agents in fibrotic lesion parenchyma (45).

Chemical shift-sequences distinguish between normal parenchyma, water, and lipid tissue, which is important in case of the high lipid content in NET metastasis (16, 46).

In general, MRI and CT have similar sensitivity and specificity in detecting the primary neoplasm and its metastases, but MRI has higher tissue resolution than CT and should be a method of choice for investigating bone metastases and it could replace CT for patients who are allergic to iodine contrast agents (10, 16, 21).

Diffusion weighted imaging (DWI) reflects tissue cellularity and cells membranes consistency which allows to detect subtle neoplastic tissue changes on a cellular level, before it could be seen as an alteration in lesion size. There is no need of contrast administration to obtain before mentioned set of information (47). DWI is highly sensitive in detecting GEP-NET liver metastases, which has been confirmed in several studies (3, 42, 44). Moreover, DWI is sensitive to post-treatment changes such as after transarterial chemoembolization (TACE) (15, 48, 49). Lu et al. observed signal changes on DWI and ADC maps within 6 months after selective internal radiation therapy(SIRT). An increase in ADC values was observed at 1 month and 6 months after SIRT. In addition, approximately 3 months after SIRT, there was a decrease in ADC values (50). DWI is the most sensitive MRI parameter in evaluation of treatment response of NET after peptide receptor radionuclide therapy (PRRT) (51).

DWI at high b-values (≥ b100) gives a low background signal from normal liver parenchyma and increases the contrast between the liver tissue and metastasis (52).

It is important to set at least three b–values to obtain apparent diffusion coefficient(ADC) maps, which could predict tumor type or grade (21). Mehmet et al. observed that ADC maps demonstrated lower values in GEP-NETs metastasis than adenocarcinoma’s maps, therefore these parameters could be used as an additional tool for assessing liver GEP-NETs metastasis which do not demonstrate strong enhancement in the arterial phase (53). Foremost, DWI in comparison to T2-weighted scans shows higher sensitivity in the detection of metastatic lesions (83% vs. 61%) (54, 55) which corresponds to the article of Sankowski et al. which confirmed that DWI and post-contrast images have higher detectability of small liver metastases than T2-weighted images (3). Additionally, DWI allows for more accurate detection of lesions around vessels and in the subcapsular parenchyma, which could be difficult to detect in routine T2-weighted sequences (55). Except for metastases detection in DWI sequences, it helps in differentiating NENs liver metastasis and hemangioma; these both lesions display hyperintensity on T2WI (16).

Intravoxel Incoherent Motion (IVIM) shows translational movements in voxel, showing both perfusion and diffusion of tissue. This phenomenon allows to depict microvasculature heterogeneity, which could translate to the assessment of treatment efficiency. It is worth mentioning that IVIM can differentiate malignant and benign lesions (56, 57). Additionally, the broader availability of MRI and no need of intravenous contrast agent administration make IVIM increasingly useful in the field of imaging of oncologic patients. Moreover, this technique decreases the need of administration of gadolinium contrast, which has the potential to deposit in the body tissue, so it could be beneficial to children, pregnant women and patients suffer from renal failure (58). Technically, the IVIM method uses the D parameter (pure diffusion coefficient), which could predict tumor grading. Hwang et al. observed that G1 tumors showed D= 1.21 × 10^−3^ mm^2^/s, compared with G2 and G3 0.95 × 10^−3^ mm^2^/s (59). Moreover, IVIM could help to differentiate vertebral metastases and atypical hemangiomas (60).

Diffusion Kurtosis Imaging (DKI) is a high advanced technique that was submitted by Jansen in 2005. It surmounts the influence of parenchymal microperfusion on diffusion restriction and is less influenced by liver parenchyma changes, such as fibrosis. It may be used when the examined lesion does not show characteristic features, like a vivid arterial enhancement, which according to Jeon SK, could be observed even in 49% of the patients’ examinations. Similarly to IVIM, DKI allows to differentiate malignant and benign lesions, as also neuroendocrine and other solid tumors found in the head of the pancreas, which is important, because of possibility of alteration of treatment (61, 62). The inconvenience of the DKI sequence is an extension of study duration, which could limit use of this technique in patients for whom keeping one position for a long time could be impossible (63).

The sensitivity of each of above mentioned radiological methods is summarized in **Table 1**.

**Table 1:** The sensitivity of each modality in detecting GEP-NENs ref (10, 12, 13, 15, 18, 26, 28–31, 52, 53).

Method	Sensitivity
**Transabdominal ultrasonography**	39% in detecting pancreatic NET
**Contrast enhanced ultrasonography**	99% in detecting liver metastases
**Endoscopic ultrasonography**	57-94% in detecting pancreatic NEN 71-94% in detecting insulinoma 74-96% in detecting pNET(head) 63% in detecting pNET(tail) and duodenal NET
**Contrast enhanced computed tomography**	63-82% in detecting pancreatic NET 20-63% in detecting insulinoma 82% in detecting liver metastases 60-70% in detecting lymph nodes metastases 58% in detecting bone metastases
**Enteroclysis/ enterography CT**	92%
**Contrast enhanced magnetic resonance and DWI **	90-95% in detecting liver metastases
**T2 sequences of magnetic resonance**	61% in detecting liver metastases
**DWI of magnetic resonance**	83% in detecting liver metastases
**Contrast enhanced magnetic resonance **	79% in detecting pancreatic NEN

## Conclusion

Despite the technical development and higher availability of radiology imaging, the diagnosis of GEP-NETs is still difficult and time consuming. The atypical biological behavior of GEP-NETs and their unspecific symptoms makes harder to give a proper diagnosis. A cooperation between clinicians, surgeons, radiologists and histopathologists is needed.

Radiologists should be aware of the possibility of false negative imaging results, e.g., in case of pNET, which could be verified by EUS. On the other hand, primary tumor of GEP-NET or its metastases could be an incidental finding during a diagnosis of unspecific abdominal pain or abdominal pain in emergency ward.

Beside detection of neoplasmatic tissue, radiology plays important role in follow – up, where the main point of interest is liver, in which metastases are found the most often. Because of vast possibilities of treatment (systemic therapies as well as targeted therapies) there is a need of introducing new ways of assessment, beyond RECIST 1.1. criteria.

In the future, this problem could be solved using advanced diffusion magnetic resonance technique like IVIM, which allows to investigate the tissue microcirculation. However, a repeatability of this techniques is low and unsystematized, so a further evaluation, in possibly broad group of patients, is necessary. Another, future direction could be a radiomics, which collects and analyzes patients data using deep learning techniques, and as a result, allows to predict the grade of tumor or treatment response (30). Considering a specific biological behavior of GEP-NETs, a combination of radiological and nuclear medicine methods seems to be promising way. Unfortunately, it is not broadly accessible nowadays.

## Author Contributions

GP-S, TB, WW, JW contributed to conception and design. GP-S, MIF, WW, KS, JW, and BM contributed to analysis and interpretation of data. GP-S, TB, MIF, KS, JW, and BM contributed to revising the article. GP-S, TB, MIF, WW, KS, JW, and BM contributed to final approval. GP-S contributed to drafting the article. All authors contributed to the article and approved the submitted version.

## Funding

The authors declare that this study received funding from Centre of Postgraduate Medical Education in Warsaw, Poland. The funder was not involved in the study design, collection, analysis,interpretation of data, the writing of this article, or the decision to submit it for publication.

## Conflict of Interest

The authors declare that the research was conducted in the absence of any commercial or financial relationships that could be construed as a potential conflict of interest.
